# Probability of causation for occupational cancer after exposure to ionizing radiation

**DOI:** 10.1186/s40557-018-0220-5

**Published:** 2018-01-31

**Authors:** Eun-A Kim, Eujin Lee, Seong-Kyu Kang, Meeseon Jeong

**Affiliations:** 1 Occupational Safety and Health Research Institute, Korea Occupational Safety and Health Agency, Ulsan, Korea; 2 Korea Occupational Safety and Health Agency, Ulsan, Korea; 3 Radiation Health Institute, Korea Hydro & Nuclear Power Co. Ltd, 172 Dolma-ro, Bundang-gu, Seongnam-si, Gyeonggi-do 13605 Korea

**Keywords:** Probability of causation, Ionizing radiation, Occupational cancer, Compensation

## Abstract

**Background:**

Probability of causation (PC) is a reasonable way to estimate causal relationships in radiation-related cancer. This study reviewed the international trend, usage, and critiques of the PC method. Because it has been used in Korea, it is important to check the present status and estimation of PC in radiation-related cancers in Korea.

**Methods:**

Research articles and official reports regarding PC of radiation-related cancer and published from the 1980s onwards were reviewed, including studies used for the revision of the Korean PC program. PC has been calculated for compensation-related cases in Korea since 2005.

**Results:**

The United States National Institutes of Health first estimated the PC in 1985. Among the 106 occupational diseases listed in the International Labor Organization Recommendation 194 (International Labor Office (ILO), ILO List of Occupational Diseases, 2010), PC is available only for occupational cancer after ionizing radiation exposure. The United States and United Kingdom use PC as specific criteria for decisions on the compensability of workers’ radiation-related health effects. In Korea, PC was developed firstly as Korean Radiation Risk and Assigned Share (KORRAS) in 1999. In 2015, the Occupational Safety and Health Research Institute and Radiation Health Research Institute jointly developed a more revised PC program, Occupational Safety and Health-PC (OSH-PC). Between 2005 and 2015, PC was applied in 16 claims of workers’ compensation for radiation-related cancers. In most of the cases, compensation was given when the PC was more than 50%. However, in one case, lower than 50% PC was accepted considering the possibility of underestimation of the cumulative exposure dose.

**Conclusions:**

PC is one of the most advanced tools for estimating the causation of occupational cancer. PC has been adjusted for baseline cancer incidence in Korean workers, and for uncertainties using a statistical method. Because the fundamental reason for under- or over-estimation is probably inaccurate dose reconstruction, a proper guideline is necessary.

## Background

In contrast to occupational injury, which has a definite cause, the causation of occupational disease is not clear in most cases. Particularly, occupational cancers cannot be distinguished from cancers occurred spontaneously in general population due to the complexity of multi-cause pathogenesis. However, the attribution of a particular cancer risk can greatly influence workers’ compensation. Therefore, quantitative risk estimation of radiation-related disease requires appropriate methods for determining probabilities under complex circumstances [1].

Using accumulated results regarding the probability of cancer following low-dose exposure to radiation and statistical modeling for assessment of the causation of ionizing radiation-induced cancer, the National Institutes of Health (NIH) in the United States first estimated the probability of causation (PC) in 1985 [2]. The International Atomic Energy Agency, International Labor Organization (ILO), and World Health Organization developed guidance on the formulation and application of PC schemes in 2010, with revisions accounting for the uncertainty in PC [3]. PC allows the attribution of cancer to occupational radiation exposure and assists decision-makers in establishing compensation schemes for occupational cancer related to ionizing radiation.

Most countries with a workers’ compensation system have adopted the occupational disease list, based on the recommendation of the ILO and European Commission, to specify compensability of a particular disease. Among the 106 occupational diseases listed in ILO Recommendation 194 [4], PC is used only for occupational cancer after ionizing radiation exposure. The United States and United Kingdom use PC as specific criteria for decisions on the compensability of workers’ radiation-related health effects [3]. The United States gave compensation to 10,479 cancer patients out of 37,155 claims (28.2%) with greater than 50% PC up to September 2015 [5]. The Compensation Scheme of Radiation-Linked Disease in the United Kingdom has considered 1496 cases since the scheme began, and 156 of these cases have resulted in successful claims based on the PC [6].

In Korea, PC was developed firstly as Korean Radiation Risk and Assigned Share (KORRAS) in 1999 and used in the Ministry of Science and Technology’s ordinance (MST; no. 2001–35: approval standard of occupational disease after exposure to radiation) in 2001 [7]. Since workers’ compensation comes under the Ministry of Labor and Employment, the MST approval standard is not a legal requirement for the compensation process. However, almost all cancer cases after ionizing radiation consider PC estimates from the Radiation Health Research Institute (RHRI) [8]. KORRAS used point estimation with uncertainty problems, while the RHRI’s revised program from 2004, Radiation Health Research Institute-Program for Estimating the Probability of Causation (RHRI-PEPC), adjusted for the uncertainty issue [7]. In 2015, the Occupational Safety and Health Research Institute (OSHRI) and RHRI jointly developed another revised PC program incorporating the recent Korean cancer incidence rate and statistical revision.

In this study, we reviewed the international trend, usage, and critique of PC estimation and the current status of the Korean PC program. In addition, we estimated PC for radiation-related cancer cases in Korea using the revised PC program.

## Methods

Research articles and official reports regarding PC of radiation-related cancer and published from 1985 onwards were reviewed. For revising the Korean PC program, we determined the statistical model for each cancer type by reviewing the latest information about cancer risk following radiation exposure. Statistical uncertainty of PC was based on the model, correction for errors in dosimetry, dependence of risk on dose and dose rate in terms of the dose-dose rate effectiveness factor (DDREF), risk transfer to the Korean population, relationship between the radiation dose and smoking history in lung cancer, and latent period. Uncertainty factors were combined using the Monte Carlo method. The PC program used organ dose assumptions. Using the revised PC program, we re-calculated the PC of compensation-related cases, which had been assessed by RHRI-PEPC since 2005.

## Results

### Statistical modeling of PC for radiation cancer

In the National Cancer Institute-Center of Disease Control (NCI-CDC) model, PC is basically a calculation of excess relative risk (ERR) as a function of radiation dose for each exposure using the following formula [9]:

$$ {\displaystyle \begin{array}{l} PC= risk\; due\; to\kern0.17em radiation\kern0.17em exposure/\left( baseline\kern0.17em risk+ risk\; due\; to\kern0.17em radiation\kern0.17em exposure\right)\times 100\%\\ {}= ERR/\left(1+ ERR\right)\times 100\\ {} Where\; ERR\left( Excess\kern0.17em relative\kern0.17em risk\right)= excess\kern0.17em risk/ baseline\kern0.17em risk\end{array}} $$

ERR is estimated after adjusting for cancer type, sex, age at exposure, attained age, and radiation dose. The sensitivity to radiation by cancer type depends on the dose coefficient in PC model which corresponds to ERR per unit dose(Sv) for exposure age(e) 30 or older and attained age(a) 50 or older for most cancer type. When we calculate the ERR/Sv at e = 30 and a = 50, the ERR/Sv in male is higher in the order of leukemia, lung cancer, thyroid cancer and cancer in kidney and other urinary organs. In female it is leukemia, cancer in kidney and other urinary organs, bladder cancer and breast cancer. Applying different age at exposure and attained age may change the order of sensitivity. The younger the age at exposure, the higher ERR. It is same for the attained age. The dose-response relationship is different according to the cancer type and the characteristics of exposure. Solid cancer and leukemia from high-linear energy transfer (LET) exposure or chronic low-LET exposure assume a linear-dose response relationship. On the other hand, leukemia from acute low-LET exposure assume the linear-quadratic dose response. In case of multiple exposures, each ERR is calculated separately and added together.

### International trend of PC for radiation cancer

Since the first PC model for radiation cancer was proposed by the NIH, there have been several models such Biologic Effects of Ionizing Radiation V [10] and United Nations Scientific Committee on the Effects of Atomic Radiation [11]. Most are based on the mortality of Japanese atomic bomb survivors without adjusting for many uncertainties. Furthermore, other limitations remain such as the extrapolation from one population to another and difference in PC according to the choice of model (additive or multiplicative model). The revised PC model proposed by the NCI-CDC in 2013 [9], based on Japanese atomic bomb survivors focused on the evaluation of the distribution of uncertainty. It adapted the random mixed model considering the basic uncertainties and was rated the most reasonable and objective model. Using the NCI-CDC model, the NCI-Interactive Radio Epidemiological Program (IREP) was developed after adjusting for the population of the United States [9] and the National Institute of Occupational Health (NIOSH) developed the NIOSH-IREP, a revision of NCI-IREP [12]. A risk model for radiation cancer based on a life span study of Japanese atomic bomb survivors is updated regularly by the Radiation Effect Research Foundation [13].

### Development and recent revision of PC for radiation cancer in Korea

The first PC program for radiation cancer in Korea was KORRAS in 1999. It used point estimation, which was revised in RHRI-PEPC by evaluation of the uncertainty using the NCI-IREP model and Korean baseline cancer incidence in 2003 [7]. In the RHRI-PEPC model, the ERR per unit radiation exposure (mSv) was estimated mainly based on the Japanese atomic bomb survivors study, for the 30 cancer types in the NCI-CDC report (Table 1) [9]. To adjust the uncertainties, the systematic and random errors of radiation exposure dose measurement were statistically revised. Uncertainty due to population transition was adjusted using the random linear combination model. In this transition, the Japanese baseline cancer incidence was from Hiroshima and Nagasaki [12] and the Korean baseline incidence was from 1993 to 1998 [14]. DDREF for chronic low-LET radiation allowed discrete distribution. For acute low-LET radiation, the starting dose at which DDREF applied was randomly decided by log-uniform distribution. In the case of lung cancer, the interaction between radiation and cigarette smoking was added as a mixed additive and multiplicative model. The relative risk of lung cancer by smoking level was obtained from an NCI-CDC report [9]. Other interaction factors such as the effect of race on skin cancer and the first full term delivery on breast cancer were not considered. The minimum latent period of cancer was assumed to be 1 year for leukemia, 2 years for thyroid cancer, and 4 years for other solid cancers. The cancer risk was phased by an S-shaped function to avoid rapid increase from 0 immediately after exposure to full value after a transition period. Therefore, minimum and maximum values were attained at 1 and 5 years for leukemia, 2 and 8 years for thyroid cancers, 4 and 11 years for most solid cancers, respectively.

**Table 1:** Cancer types for Radiation Health Research Institute-Program for Estimating the Probability of Causation

	Cancer Location	ICD-9 code	ICD-10 code
1	Oral cavity and pharynx	140–149	C00–14
2	Esophagus	150	C15
3	Stomach	151	C16
4	Colon	153	C18
5	Rectum	154	C19–20
6	Liver	155.0	C22
7	Gall bladder	155.1156	C23–24
8	Pancreas	157	C25
9	Trachea, bronchus and lung	162	C33–34
10	Nasal cavity, larynx, and others	160,161,163–165	C30–32,C37–38
11	Bone	170	C40–41
12	Connective tissue	171	C47,C49
13	Malignant melanoma	172	C43
14	Non-melanoma skin-basal cell carcinoma	173 (all combined)	C44
15	Non-melanoma skin-squamous cell carcinoma	173 (all combined)	C44
16	Breast-female	174	C50
17	Breast-male	175	C50
18	Ovary	183	C56
19	Female genitalia except ovary	179–182,184	C51–55,C57–58
20	All male genitalia	185–187	C60–63
21	Bladder	188	C67
22	Kidney and other urinary organs	189	C64–66,C68
23	Eye	190	C69
24	Brain and nervous system	191,192	C70–72
25	Thyroid	193	C73
26	Other endocrine glands	194	C74–75
27	Other and ill-defined sites	195	O&U
28	Lymphoma and multiple myeloma	203	C82–85,C88,C90,C96
29	Leukemia, except chronic lymphocytic leukemia	204–208, less 204.1	C91–95 except C91.1

ICD: International Classification of Diseases

In 2015, OSHRI and RHRI jointly revised the RHRI-PEPC to Occupational Safety and Health (OSH)-PC for the assessment of uncertainties and baseline cancer incidence in Korea. Among the NCI-CDC [9], a radiation risk assessment tool for lifetime cancer risk projection (RadRAT) program [15], and Japan atomic bomb survivor [16] models, the NCI-CDC model included a large number of cancer types and an uncertainty evaluation method for each cancer. Finally, the OSH-PC program included risk models for 29 cancer types (Table 1) from RHRI-PEPC except digestive system cancers. Among the solid cancers in Table 1, bone cancer, connective tissue cancer, eye cancer, endocrine glands cancer except thyroid cancer, and other, ill-defined cancers do not have individual cancer risk models. For these cancers, the residual solid cancer model from NCI-CDC was adopted. Male breast cancer used the female breast cancer model. For malignant melanoma, the non-melanoma skin cancer model was used. While NIOSH-IREP and RHRI-PEPC used the age-standardized baseline cancer incidence rate to assess population transfer uncertainty, OSH-PC adopted the age-specific cancer incidence rate for a 5-year interval and therefore gave us more accurate PC values for each case. It is well known that lung cancer risk depends on the interaction of radiation and smoking. If the worker is a smoker, the contribution of radiation to his lung cancer is lower than in non-smoker. In the new PC model we reflected the smoking-related adjustment factors in Korea for each lung cancer type (squamous cell, adenocarcinoma, small cell, other types) and the factors were derived using lung cancer relative risks by smoking category in Korea [17]. Differences between RHRI-PEPC and OSH-PC were summarized in Table 2.

**Table 2:** Summary of Differences between RHRI-PEPC and OSH-PC

Items	RHRI-PEPC	OSH-PC
Cancer types	30	29 (exclude a category of digestive system cancers combined)
Baseline cancer incidence rate	Use a value as age-standardized rate (ASR) published by IARC [14]	Use Korean age-specific incidence rate in the year of diagnosis
Lung cancer type	1 category for lung cancer	4 categories by cell type (squamous cell, adenocarcinoma, small cell, other types)
Lung cancer relative risk for smoking-related adjustment	Use relative risk by smoking level in the U.S. population	Use Korean lung cancer relative risk by smoking level [17]

### PC of cancer cases after radiation exposure in Korea

In the 10 years since 2005, 16 claims of workers’ compensation for cancers after radiation exposure used PC (Table 3). Half of these were lymphohematopoietic cancers (7 leukemias and 1 lymphoma). The remainder included three thyroid cancers, and one case each of breast cancer, auditory canal cancer, rectal cancer, multiple cancer (gastric and pancreatic), and unknown origin cancer. Leukemia cases consisted of three lymphatic leukemia and four myeloid leukemia (three myelocytic, one myelomonocytic, and one myeloblastic) cases. Seven cases involved health care industry workers such as a radiologist, radiological technologists, and a dental nurse. Others worked in nuclear power plants (3 cases) as operators or in radioactive waste treatment, non-destructive tests (3 cases), the semiconductor industry as maintenance workers (2 cases), and sales of medical devices such as the installation of X-ray instrument (Table 3).

**Table 3:** Cancer cases after radiation exposure in Korea (2005–2014)

No	Dx Year	sex	Industry	Job	Disease	1st Exp	Dx age	DR	CDose (mSv)	Decision	Latency	PC1		PC2	
												95th	99th	95th	99th
1	2005	male	Nuclear power plant	Radioactive Waste Treatment	Squamous Cell Carcinoma in the External Auditory Canal	34	60	16y	284	NA	26y	0	16.7	6.9	13.3
2	2004	male	Service	NDT	Carcinoma of unknown primary site	30	46	9 m	1870	A	16	61.7	67.3	50.7	62.4
3	2009	male	Healthcare	Radiologist	Thyroid papillary cancer	28	36	8y8m	245	NA	8y8m	21.9	33.3	21.9	33.3
4	2005	male	Nuclear power plant	Operator	Gastric cancer/pancreatic cancer	19	53	21y5m	21.4	NA	24y	6.2	10	4.6	7.2
5	2006	male	Nuclear power plant	Operator	Acute myelocytic leukemia	23	34	11y	16.5	NA	11y	16	20.1	24.4	29.3
6	2010	male	Semiconductor	Maintenance	Chronic myelomonocytic leukemia	24	37	13	34.7	NA	13y	34.9	40.3	35.8	41.3
7	2011	male	Service	X-ray Installation Instrument	Anaplastic large cell lymphoma	30	39	6y	265	A	9y	8.1	15.7	4	7.2
8	2009	female	Semiconductor	Operator	Breast Cancer	19	33	4y8m	16.4	NA	13y	9.2	12	5.6	7.2
9	2011	male	Healthcare	Radiologist	Chronic myelocytic leukemia	24	45	22y	204	A	22y	60.4	65	55	58.9
10	2011	male	Machine manufacturing	Maintenance	Acute lymphocytic leukemia	27	56	47d	1.7	NA	29y	1.5	2.3	0.9	1
11	2011	male	Service	NDT	Acute lymphocytic leukemia	27	37	10y1m	51	A	10y1m	48	53.8	45.3	50.8
12	2011	male	Service	NDT	Acute myeloblastic leukemia	37	42	2 m	7.2	NA	15	10.6	12.8	14.2	17.5
13	2011	male	Healthcare	Radiologist	Acute lymphocytic leukemia	35	45	9 m	8.3	A	10y	7.6	9.1	6.5	7.5
14	2012	female	Dental clinic	Radiography	Thyroid papillary cancer	38	50	6y5m	93.4	NA	12y	8.0	14.8	8.0	14.8
15	2013	male	Healthcare	Radiological Technologist	Rectal cancer	25	52	26y	72.2	NA	26y	1.2	1.9	1	1.6
16	2013	female	Healthcare	Radiological Technologist	Thyroid papillary cancer	43	28	16y	3.8	NA	16y	0.7	1.2	0.7	1.2

Dx year: year of diagnosis, 1st exp.: age at first exposure, latency: years from first exposure to diagnosis, NDT: non-destructive test, Dr.: exposure duration in year, CDOSE: cumulative dose in mSv, y: year, m: month, decision: result of workers’ compensation process, A: accepted, NA: not accepted, PC1: Probability of Causation assessed by Radiation Health Research Institute-Program for Estimating the Probability of Causation, PC2: Probability of Causation assessed by Occupational Safety and Health-Probability of Causation

Assessment conducted using RHRI-PEPC when the claim was filed (PC1), and by OSH-PC after revision of the PC program (PC2) (Table 3). Because the RHRI-PEPC and OSH-PC dealt with all types of leukemias except chronic lymphocytic leukemia as leukemia, the PC of the 7 cases were assessed as leukemia (Table 3). The cumulative radiation exposure dose for the leukemia cases was 1.7–204 mSv, and the upper 99th confidence limits of PC1 and PC2 were 2.3–65% and 1–58.9%, respectively (Table 3). Workers’ compensation was offered in two leukemia cases with more than 50% PC in the 99th confidence level. A radiologist with 9.1% of PC at the 99th confidence level was accepted for workers’ compensation because his cumulative radiation exposure dose was not believed to correctly reflect the actual exposure. Considerations included inappropriate protective glove during the procedure, higher cumulative dose of co-workers, and testimony of co-workers that they used to work without a film badge. Work-relatedness of a leukemia case was not decided because of lack of objective exposure level data (Table 3). A non-Hodgkin’s lymphoma case with 15.7% PC at the 99th confidence level was accepted for compensation because the exposure dose was believed to be underestimated.

The PCs of three thyroid cancer cases at the 99th confidence interval were 1.2%–33.3% with 3.8–245 mSv of cumulative dose. Workers’ compensation was not given for any of these cases, while a case of carcinoma of unknown primary site was accepted with high PC (67.3%) and cumulative dose (1870 mSv). The PC of a squamous cell carcinoma in the external auditory canal was analyzed under ‘the rest of unclassified cancers or unclear cancer’ category, and was shown to be 16.7% with 99th confidence intervals and not accepted for compensation. The PC of rectal and breast cancer was lower than 1.9% and 12%, respectively, with 72.2 mSv and 16.4 mSv dose, respectively. The PC of multiple cancers assessed as a multiple effect (gastric cancer + pancreatic cancer) was 10% at the 99th confidence level (Table 3).

Looking at the three cases (number 2, 9, 11) although case 2 was exposed to more radiation dose, case 2 (1870 mSv) and case 9 (204 mSv) have similar PC values. This is presumably due to the fact that leukemia has a higher radiation risk than ‘the rest of unclassified cancers or unclear cancer’ and that the age at first exposure of case 9 (24 yr) was younger than case 2(30 yr). Leukemia in case 11 (51 mSv) has a lower exposure than case 9, but the PC value is quite high. The reason is presumably because he was exposed to radiation in a relatively short period (10y1m).

PC2 assessed by OSH-PC was higher than PC1 assessed by RHRI-PEPC in three cases (number 5, 6, 12). PC2 in other cases were lower than PC1 except three cases with the same PC (number 3, 14, 16). The highest variation was seen for a carcinoma of unknown primary site (number 2). In case of the 95th and 99th PC, the highest variation was observed for acute myelocytic leukemia (number 5, Table 3). In thyroid cancer, PC1 and PC2 showed the same results because ERR was transferred independently based on baseline cancer incidence.

The exposure dose of some cases, especially number 3 and 13 based on the estimation based on the statements by the patient and their co-workers, the PC of them might be distorted from actual level.

## Discussion

In Korea, PC assessment has been used in the workers’ compensation process through the epidemiologic investigation of OSHRI, which referred to the Korea Workers’ Compensation and Welfare Service [18]. OSHRI and RHRI jointly estimated the PC of cancer cases in the epidemiologic investigation of workers’ cancer after exposure to ionizing radiation. PC provides important evidence regarding whether radiation has a substantial effect. However, exceptions were made in some cases for low PC (lower than 50% at the 99th confidence interval) because of possible underestimation of the exposure dose. A possible explanation for the underestimation of radiation dose is the low wearing rate for film badges [19, 20]. Another possibility is that the monitoring system assigned 0 values to the value having under the detection limit of the assessment system [21]. PC estimation requires an accurate dose estimate for more reliability. To reduce underestimation or overestimation of radiation exposure, a reasonable dose reconstruction guideline should be prepared for the nationwide working environment. In the Unites States, NIOSH applies the radiation dose reconstruction method under the energy employees occupational illness compensation program act of 2000 [22].

Since ILO added radium and other radioactive substances as well as X-rays to the occupational list of the Workmen’s Compensation (Occupational Diseases) Convention number 42 (C042) in 1934 [23], ionizing radiation has constantly been on national and international occupational disease lists. In 1964, the item’s name was changed to ‘ionizing radiation’ in Schedule I of the Occupational Disease List of the Injury Benefits Convention number 121 [24]. The current ILO occupational disease list in ILO Recommendation 194, which has been adopted by the majority of ILO member states, contains 106 occupational diseases [4]. Thanks to the long history of cancer risk research, especially the experience of the Japanese atomic bomb cohort, PC could have been developed for ionizing radiation.

For most cancer types, the difference between PC1 and PC2 values was mainly due to the population transfer method. PC1 used age standardized rate (ASR) during 1993–1998 [14] for Korean baseline cancer incidence rate but PC2 used age-specific cancer incidence rate in the year of diagnosis.

Because the PC model used the cancer risk model based on ERR from the Japanese atomic bomb survivor cohort, there are statistical uncertainties and a measurement error in the dose data. In addition, extrapolation between different populations, and DDREF must be considered when the model is applied to the low dose and low dose rate exposure groups. The interaction effect of radiation and cigarette smoking and interpersonal difference of the latent period can have uncertainties. PC model should be estimated considering all the uncertainties and needs distribution and the confidence interval rather than point estimation. OSH-PC program gives the results based on distribution and confidence interval after adjusting all these uncertainties.

With extension of the follow-up period for Japanese atomic bomb survivors and more research on the risk of radiation-related cancer, better statistical models could develop.

In OSH-PC, all types of leukemia except chronic lymphocytic leukemia (CLL) were considered as a group. PC could not been estimated separately for acute myelogenous, acute lymphocytic, and chronic myelocytic leukemia because each baseline incidence rate was not able to use in Korea. In 2013, NIOSH-IREP incorporated the CLL risk model as a group of lymphoma and multiple myeloma because CLL appears etiologically and clinically to be a lymphoma [25] and a risk model for CLL has been developed similar to that for lymphoma and multiple myeloma [26]. In OSH-PC, CLL was excluded from the PC calculation because its association with radiation exposure was not specified clearly even in Japanese atomic bomb cohort and is still controversy.

Greenland and others [27–29] have argued that PC is a logically flawed concept and therefore unsuitable for the adjudication of compensation claims in possible cases of radiation-related cancer. They argued that PC based on epidemiologic data which does not consider biologic mechanism, might be unavoidable the uncertainties. The NCI-CDC working group concluded that the argument may have theoretical merit but, as a practical matter, is unpersuasive in the light of current information about radiation-related risk [9]. Even though PC has limitations, its use seems to be inevitable in estimating the radiation-related cancer risk, especially for the worker’s compensation process, keeping in mind that accurate dose reconstruction reflecting the workplace exposure is more important than PC itself.

## Conclusion

PC is one of the most advanced tools for estimating the causation of occupational cancer. Despite issues of uncertainty, PC for Korean workers has been adjusted for the baseline incidence of cancer, and recently statistical methods have been used to adjust for the uncertainties. Because the fundamental reason of under- or over-estimation is probably inaccurate dose reconstruction, a proper guideline is necessary.

## Abbreviations

ASR: Age standardized rate
CLL: Chronic lymphocytic leukemia
DDREF: Dose-dose rate effectiveness factor
ERR: Excess relative risk
ILO: International Labor Organization
KORRAS: Korean Radiation Risk and Assigned Share
LET: Linear energy transfer
MST: Ministry of Science and Technology’s ordinance
NCI-CDC: National Cancer Institute-Center of Disease Control
NCI-IREP: NCI-Interactive Radio Epidemiological Program
NIH: National Institutes of Health
NIOSH: National Institute of Occupational Health
OSH-PC: Occupational Safety and Health-PC
OSHRI: Occupational Safety and Health Research Institute
PC: Probability of causation
RadRAT: A radiation risk assessment tool for lifetime cancer risk projection
RHRI: Radiation Health Research Institute
RHRI-PEPC: RHRI-Program for Estimating the Probability of Causation
